# A Comparison Between Silodosin and Tamsulosin for Medical Expulsive Therapy of Distal Ureteric Calculus

**DOI:** 10.7759/cureus.47008

**Published:** 2023-10-14

**Authors:** Atif Abdullah, Yogendra Basoo Gupta, Sudhakaran Selvaraj, Ramesh Ganapathy, Ananda Kumar Ilangovan, Senthilkumar Sivalingam, Srikala Prasad

**Affiliations:** 1 Urology, Chengalpattu Medical College Hospital, Chengalpattu, IND

**Keywords:** analgesics, stone expulsion time, tamsulosin, silodosin, medical expulsive therapy

## Abstract

Introduction: Medical expulsive therapy (MET) is an established treatment option for distal ureteric stones. Tamsulosin, a selective alpha-1 blocker, has been used for MET with good results, while silodosin, a more selective alpha-1a blocker, is more effective than tamsulosin for MET. Thus, this study aimed to compare the efficacy of silodosin with tamsulosin.

Methods: This prospective randomized study was conducted at the Department of Urology, Government Chengalpattu Medical College Hospital, Tamil Nadu, India. Eighty patients who presented with ureteric colic and were radiologically diagnosed with distal ureteric calculus of size <10mm were included. Participants in the silodosin group received tablet silodosin 8mg OD until the passage of the stone, not more than two weeks, and analgesics as per demand. And participants in the tamsulosin group received tablet tamsulosin 0.4mg OD until the passage of the stone, not more than two weeks, and analgesics as per demand.

Results: A total of 80 patients were included in the study. Forty patients in the silodosin group and forty patients in the tamsulosin group were included. In the silodosin group, out of 40 patients, 38 expelled the calculus. In the tamsulosin group, out of 40 patients, 28 expelled the calculus. The silodosin group had a significantly higher rate of expulsion, with a p-value of 0.003. Stone expulsion time was shorter in the silodosin group when compared with the tamsulosin group (10.15 vs. 13.4 days). Analgesic usage during medical expulsive therapy was lower in the silodosin group (5.68 vs. 8.4). We observed significant differences in comparing the outcome, stone expulsion time, and analgesic requirement between the silodosin and tamsulosin groups. We observed no significant difference between the groups for age-wise and gender-wise comparisons. Furthermore, non-expulsion of calculus in four patients and pain in eight patients were the reasons for intervention in the tamsulosin group. The reason for intervention in the silodosin group was the non-expulsion of calculus in two patients.

Conclusion: Using silodosin for MET of distal ureteric calculus, we found to have a better stone expulsion rate, early expulsion time, and reduced analgesic requirement.

## Introduction

Urolithiasis is a significant clinical and financial burden on modern healthcare systems. According to international epidemiological data, kidney stone disease (KSD) is rising, with a lifetime prevalence of roughly 14% and a recurrence rate of 50% or more within 10 years. Even though the average age of KSD patients is between 40 and 60 years, there is an alarming increase in the number of children diagnosed with the condition. Despite a steep rise in minimally invasive procedures, medical expulsive therapy (MET) is an established treatment option for distal ureteric stones [1]. Passage rates of stones measuring < 5mm range from 71 to 98%, and stones measuring 5mm to 10mm range from 25% to 53% [2].

Spontaneous passage factors are location, size, and number; ureteric spasm; mucosal oedema or inflammation; and ureteric anatomy. Stone-induced ureteric spasms impede calculi evacuation. Alpha-blockers block alpha-1 receptors, which are abundant in the distal third of the ureter. This blockage causes inhibition of basal smooth muscle tone and hyperperistalsis while maintaining tonic propulsive contractions [3,4]. Tamsulosin, a selective alpha-1 blocker, has been used for MET with good results. Silodosin, a more selective alpha-1 blocker, is more effective than tamsulosin for MET. Thus, this study aimed to evaluate the efficacy of alpha-blockers for the expulsion of distal ureteric calculus and to compare the efficacy of silodosin with tamsulosin.

## Materials and methods

**Figure 1:**
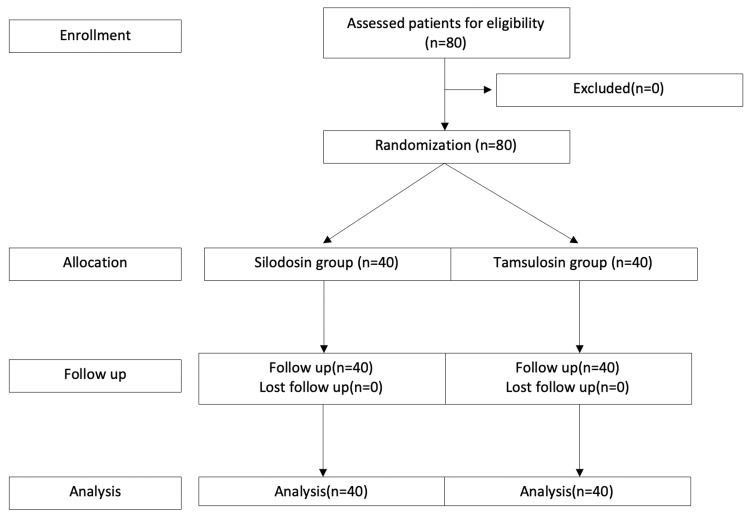
CONSORT flow diagram of the trial CONSORT: Consolidated standard of reporting trial

This prospective randomized study was conducted between September 2020 and May 2021 at the Department of Urology, Government Chengalpattu Medical College Hospital. The study lasted nine months, and a total of 80 patients were included. This study received approval from the Institutional Ethical Committee, Chengalpattu Medical College (approval no. IEC-CMC/Approval/5945/2020) and is registered in the Clinical Trials Registry of India (CTRI) (no. CTRI/2020/11/029244). Patients who presented with renal colic and were radiologically diagnosed with distal ureteric calculus of size <10mm were included. Stones larger than 10 mm, patients with urosepsis, multiple calculi, severe hydroureteronephrosis, patients who had taken alpha-blockers, pregnant women, patients who had undergone endoscopic procedures for calculus, patients with a history of spontaneous stone expulsion, patients with ureteral stricture, and patients who didn't provide consent were excluded from the study. A block randomization technique was used to randomize patients (Figure 1).

The silodosin group received tablet silodosin 8mg once daily (OD) until the passage of the stone, not more than two weeks, and analgesics as per demand. The tamsulosin group received tablet tamsulosin 0.4mg OD until the passage of the stone, not more than two weeks, and analgesics as per demand. Patients were reviewed every week until the passage of calculus, or up to four weeks. Factors analyzed were age and sex, calculus passage rate, calculus passage time, patients requiring intervention, the reason for intervention, and analgesic requirements. The primary endpoint of the study was the passage of calculus. The MET was discontinued in cases of intractable pain or urosepsis. The efficacy was evaluated using the student t-test and chi-square test with a 5% significance level.

## Results

The study involved 80 patients in total. Forty patients were in each group, i.e., the silodosin group and the tamsulosin group. No significant difference was observed between the groups on age-wise and gender-wise comparisons. Moreover, we compared the stone size, outcomes, expulsion time, and analgesic requirements between the silodosin and tamsulosin groups. We observed significant differences in the outcome, stone expulsion time, and analgesic requirement between the two groups (Table 1).

**Table 1:** Comparison of various parameters between silodosin and tamsulosin groups

Variables	Tamsulosin group (n=40)	Silodosin group (n=40)	p-value
Age (years)	40.9 (29.1-52.19)	36.28 (22.7-49.86)	0.102
Male	16	22	0.2
Female	24	18	
Calculus size (mm)	6 (4.3-7.6)	6.55 (4.9-8.1)	0.13
Outcome: Failed expulsion	12	2	0.003
Stone expulsion time (in days)	13.4 (7.1-19.7)	10.15(5.25-15.05)	0.012
Analgesic requirement	8.4 (3.5-13.3)	5.68 (3.5-7.7)	0.002

**Figure 2:**
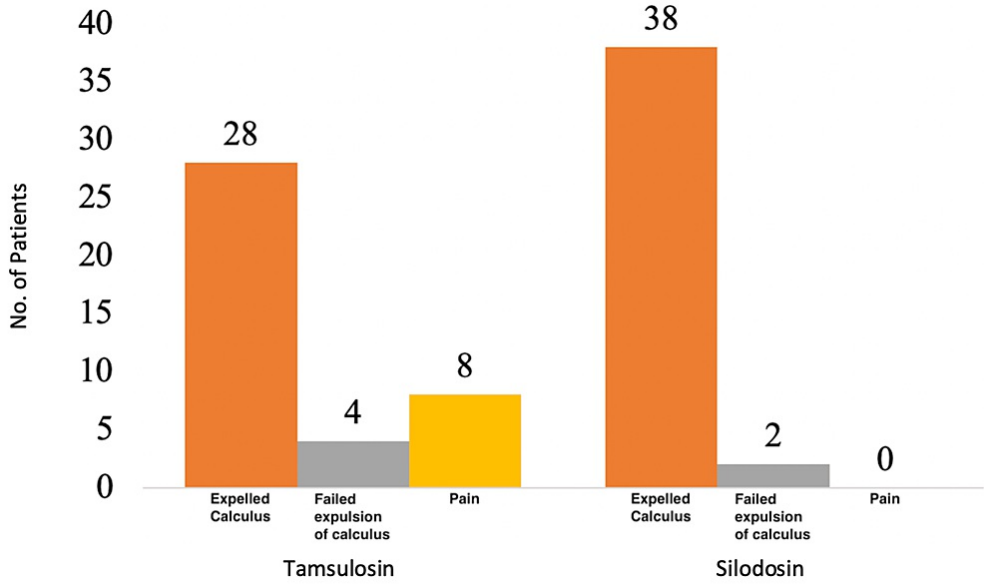
Reason for intervention in the silodosin and tamsulosin groups

Further, we studied the reason for intervention between the silodosin and tamsulosin groups, as shown above in Figure 1. We observed that non-expulsion of calculus and pain were the reasons for intervention in four and eight patients, respectively, in tamsulosin group 1, whereas non-expulsion of calculus was the only reason for intervention in two patients in silodosin group 2 (Figure 2).

## Discussion

Ureteroscopy and shockwave lithotripsy continue to be the most effective treatments for distal ureteric stones; nevertheless, they are costly and not without risk. However, spontaneous stone evacuation can occur in up to 50% of cases. Problems such as ureteric colic and urinary tract infections can occur. The use of adjuvant drugs like tamsulosin for distal ureteric stones has helped reduce discomfort and complications and increase the pace of stone clearance [1].

The most common receptors in the distal ureter are the alpha-1A- and 1D-adrenoceptors. Activating these alpha-1-adrenoceptors enhances ureteric peristalsis frequency and the force of ureteric contractions. However, blocking these receptors reduces baseline ureteric tone, peristaltic frequency, and amplitude. Resulting in a drop in intraluminal pressure while increasing the pace of urine transport [1].

Various adjuvant medications reduce complications and favor the expulsion of stones [5,6]. Tamsulosin is a selective alpha-1 blocker with a 10-fold greater affinity for alpha-1a and alpha-1d adreno-receptor subtypes. Silodosin is a more selective alpha-1 blocker with a 50-fold greater affinity to the alpha-1a adreno-receptor subtype and fewer cardiovascular side effects [7].

Gupta et al. [8] compared silodosin and tamsulosin as METs for distal ureteric calculus and found the silodosin group to have a higher clearance rate (82% vs. 58%). Similar results were found in studies conducted by Elgalaly et al. [9] (83% vs. 57%) and Kumar et al. [10] (83.3% vs. 64.4%). Our study found that the silodosin group's stone expulsion rate was higher (95% vs. 70%, p = 0.003). However, Imperatore et al., in their study, found no significant difference in stone clearance rate (88% vs. 84%) [11]. Yuksel et al. found that silodosin improved the stone expulsion rate but didn't reduce the renal colic episode or analgesic dosage [12]. In our study, analgesics required due to renal colic were significantly less in the silodosin group (5.6 vs. 8.4, p = 0.002), which was not the case in other studies.

Itoh et al., in their study, found that the stone expulsion time in the silodosin group was 11.33±8.31 days when compared to 21.0±9.9 days in the tamsulosin group [13]. Similarly, our study had a shorter stone expulsion time in the silodosin group (10.15 vs. 13.4, p = 0.012).

A systematic review and meta-analysis done by Huang et al. reported that silodosin had a better expulsion rate, shorter expulsion time, and fewer pain episodes when compared with tamsulosin [14]. They also found that silodosin had higher rates of abnormal ejaculation than tamsulosin; however, it was not statistically significant. Another meta-analysis of randomized control trials done by Liu et al. found no significant difference between silodosin and tamsulosin in terms of expulsion time, analgesic use, or retrograde ejaculation [15].

There was no treatment discontinuation among patients due to the adverse effects of the drugs. Orthotopic hypotension was reported in one (2.5%) patient in the silodosin group and three (7.5%) patients in the tamsulosin group, which was not statistically significant. This study had some limitations: a smaller sample size and other side effects of drugs like retrograde ejaculation were not assessed in depth.

## Conclusions

We found that silodosin when used as medical expulsive therapy for distal ureteric calculus of size less than 10mm, had a better stone expulsion rate when compared to tamsulosin. It also reduced the stone expulsion time significantly. Our study also showed decreased analgesic usage with silodosin. Hence, we suggest silodosin can be used for faster and less painful expulsion of distal ureteric calculus that is less than 10mm in size. However, large-scale studies are needed to confirm its efficacy further.
